# Knowledge and social beliefs of malaria and prevention strategies among itinerant Nomadic Arabs, Fulanis and Dagazada groups in Chad: a mixed method study

**DOI:** 10.1186/s12936-022-04074-0

**Published:** 2022-02-19

**Authors:** Azoukalné Moukénet, Beackgoubé Honoré, Helen Smith, Kebféné Moundiné, Wang-Mbe Djonkamla, Sol Richardson, Makido Dormbaye, Ngarkodje Ngarasta, Ibrahima Seck

**Affiliations:** 1 Cheikh Anta Diop University, Dakar, Senegal; 2 Malaria Consortium Chad, N’Djamena, Chad; 3 Independent Consultant, International Health Consulting Services Ltd, Merseyside, UK; 4 UNICEF Chad, N’Djamena, Chad; 5 Malaria Consortium, London, UK; 6 National Malaria Control Programme, N’Djamena, Chad; 7 University of Ndjamena, N’Djamena, Chad

**Keywords:** Knowledge, Social beliefs, Nomads, Chad, Malaria, Prevention

## Abstract

**Background:**

Nomadic populations in Chad are at increased risk of contracting malaria because of their lifestyle. Being highly mobile they are often excluded from disease control programmes, and access to preventive measures and treatment is more difficult. Effective malaria control interventions take account of local modes of transmission, patterns of care-seeking behaviour and community perceptions of cause and prevention practices. There is currently little information about malaria knowledge and perceptions among nomadic groups in Chad, or their awareness of malaria control interventions and this study sought to address this knowledge gap.

**Methods:**

A mixed methods study, including a cross-sectional survey with men and women (n = 78) to determine the level of knowledge and use of malaria prevention strategies among Arabs, Peuls and Dagazada nomadic groups. Three focus group discussions were conducted with women to explore their representation of malaria and knowledge of preventive methods. Key informant interviews were held with leaders of nomadic groups (n = 6) to understand perception of malaria risk among itinerant communities.

**Results:**

Nomads are aware of the risk of malaria, recognize the symptoms and have local explanations for the disease. Reported use of preventive interventions such as Seasonal Malaria Chemoprevention (SMC) for children and Intermittent Preventive Treatment (IPT) of malaria in pregnancy was very low. However, 42.3% of respondents reported owning at least one LLIN and 60% said they slept under an LLIN the night before the survey. In case of a malaria episode, nomads seek clinicians, informal drug sellers in the street or market for self-medication, or traditional medicine depending on their financial means. Interviews with nomad leaders and discussions with women provide key themes on: (i) social representation of malaria risk and (ii) social representation of malaria and (iii) perspectives on malaria prevention and (iv) malaria treatment practices.

**Conclusion:**

The nomadic groups included in this study are aware of risk of malaria and their level of exposure. Local interpretations of the cause of malaria could be addressed through tailored and appropriate health education. Except for LLINs, malaria prevention interventions are not well known or used. Financial barriers lowered access to both mosquito nets and malaria treatment. Reducing the barriers highlighted in this study will improve access to the healthcare system for nomadic groups, and increase the opportunity to create awareness of and improve uptake of SMC and IPT among women and children.

**Supplementary Information:**

The online version contains supplementary material available at 10.1186/s12936-022-04074-0.

## Background

In Chad, malaria is endemic and responsible for a significant proportion of morbidity and mortality; particularly among children aged under five. In 2017, the malaria prevalence in the general population as that in the young children of Chad were 40.9%. In the Sahelo-Saharian region of the country the malaria prevalence were 7.7% and 8.8% respectively in general population and young children [1]. In health facilities, malaria remains the main cause of consultation and hospitalization [2]. The disease profile of nomads is known to differ from settled populations [3–7]. In nomadic populations, malaria prevalence reaches 3.3% among Arab groups and near to 30% among the Fulani group living on the shore of Lake Chad (located in the Sahelo–Saharian region) in the dry season [8, 9]. The humid environment by the lake means mosquito density is high and nomads experience malaria episodes more frequently [6]. After living temporarily in the south, nomadic groups often tend to acquire malaria and in the north they are treated before the first cases of severe malaria are detected in the sedentary population [10]. In recent years, deadly malaria epidemics have developed among nomad populations, often attributed to lower immunity to malaria [11, 12]. There is some evidence that global warming has increased mobility of nomadic groups; increased movement between areas of high and low malaria transmission can result in malaria being brought into areas that were previously malaria-free, and increased vulnerability of nomads who move from low to high transmission areas [13].

The implementation of effective interventions (e.g. vector control and chemoprevention), and access to diagnostic tests and treatment, has made it possible to reduce the mortality rate from malaria worldwide by 47% between 2000 and 2013 [14]. The distribution of long-lasting insecticide-treated mosquito nets (LLINs) is one of the most promising [15] and cost-effective vector control strategies for children [16] and pregnant women [17]. In Chad, LLINs are primarily distributed through mass campaigns every three years and routinely at health facilities for pregnant women during their first Antenatal Consultation (ANC) and for children from 0 to 11 months during routine vaccination. Intermittent preventive treatment have also been shown to be effective in preventing malaria for pregnant women [18]. Seasonal Malaria Chemoprevention has been proven to be effective for children 3–59 months old in the Sahel region of Africa [19–25]. In Chad, pregnant women from the fourth month of pregnancy receive intermittent preventive treatment in pregnancy (IPT) during their ANC in health facilities.

A concern in Chad and other Sahelian countries is that health programmes, including malaria prevention and control interventions, often do not reach nomads [26, 27]. The situation seems to be the same for malaria control interventions for which strategies and protocols are not adapted for mobile populations [26]. For example, in Chad, nomadic groups are not integrated in the census for SMC [27] and LLINs mass distribution campaigns have no strategy for nomadic enumeration. Anecdotal evidence suggests the door-to-door strategy used for SMC is not compatible with itinerant families unless members of nomadic communities are used as distributors [26]. In addition, nomadic women tend not to receive IPT when they face geographical barriers to accessing antenatal clinics, or feel excluded from services available in the locations where they take up temporary residence [3, 10]. However, little is known about the level of awareness and knowledge of malaria and perceptions of malaria control interventions among nomadic groups in Chad. The ability of the malaria control programme to serve the nomadic population depends on better understanding of nomadic groups’ knowledge of malaria and its mechanism of transmission, as well as perceptions of prevention and treatment interventions and the barriers and facilitators to uptake by highly mobile groups [23–26]. Thus, this study aimed to assess the level of knowledge and explore perceptions of malaria and control interventions among the main nomadic groups in Chad. The motivation for conducting this study at this time was to inform policy decisions relating to integrating nomadic populations into the health system in order to make malaria prevention and treatment services more accessible to these often excluded groups.

## Methods

### Study site

Nomads represent 3.5% of the Chadian population [28]. They own 75% of the national herds and the trade of their animals contributes 12.5% to the Gross Domestic Product [29]. Nomads live mainly between the Saharan and Sahelian zones. In the Sahelian zone, the main nomadic groups who reside temporarily are the Dazagada (from the Toubou group), the Arabs and the Fulani [30]. The Dazagada live further south within the Sahelian zone, in areas around Borkou and further west as far as Lake Chad, Arabs generally live on the shores of Lake Chad during the dry season and the Fulanis are found in the provinces of Chari Baguirmi, Lake and Kanem [31]. Following climatic [32], economic [33] and political [34] changes over the past decades, a considerable increase in pastoral mobility has been recorded in the Sudanian zone [35]. These transhumant nomads migrate from the Saharan zone at the end of rainy season (November–January) to reach the Sudanian zone (March–April) and leave the Sudanian zone at the beginning of the rainy season (mid–April–May). The migration is mainly led by search for pastures and water. It is seasonal, cyclic and well-organized following a precisely calendar with four periods: *darat* (October), *sef* (November–May), *rachach* (June) and *Karif* (July–September). Local migrations happened in *darat* and *rachach* while provincial, national and international migration happened in *sef* and *karif* [36]. Generally the social organization of nomad consist of *beyt* or family (monogamy or polygamy) of 5–15 persons depending on one main hut. Many family with a common ancestor can live in one camp to constitute a *khashimbet* or lineage. A *khashimbet* can have 500–1000 persons with a one leader of camp. Many *khashimbet* can have one common mythic ancestor, they belong to the same *khashimbet kebir* or clan/ethnic group with up to 5000 persons. The *khashimbet kebir* have one representant of group as leader. All clan or ethnic groups belong to the same ancestor constitute a *nafar* or *qabila* with 10–50 clan groups [36].

The study was conducted among Fulani, Arabs and Dazagada nomadic communities in the provinces of Hadjer Lamis and Chari Baguirmi in the Sahelian zone of Chad, and Moyen Chari in the Sudanian zone. These three groups were included in the study because they are the main nomadic ethnic groups in Chad and they travel across all epidemiological malarious areas in Chad, which can allow to capture diversity in malaria experiences. For the qualitative part of this study two camps from each nomadic groups were retained. To integrate the view of nomads adopting a sedentary lifestyle, Fulani groups of Korbol (Moyen Chari) were retained. Regarding the quantitative part of the study, camps were divided into three groups including the Arabs (11 camps in Dourbali and 6 in Massenya), the Fulani (6 camps in Dourbali) and the Dazagada (17 camps in Massaguet). All camps retained for qualitative part of the study were surveyed.

### Study design

A mixed methods study was conducted using focus group discussions (FGD) with women (mothers) and interviews with community leaders (camp leaders), and a cross-sectional survey of nomad men and women.

### Participants

Due to the role of women to provide care to children and their experience related to IPT, only elder and younger women who are mothers were included. For the interviews, nomadic leaders (all men) who have a global view on issues face by their community and have the authority to decide the itinerary and time to move were retained. Regarding the survey, any person in the household (man or woman) willing to answer and agree to participate to the survey was included. Participation in the study required informed consent and being older than 18 years old.

### Data collection

#### Cross sectional survey

A structured electronic questionnaire (see Additional File 3) based on Peto et al. [37] was implemented using Magpi software v6.2.1 [38], and was administered offline with responses uploaded to the server once WiFi connection was available. A sample size was calculated using a prevalence of malaria among nomads assuming a worst scenario (30%), 90% Confidence Interval (90% CI), 9% margin of error and contingency for a non-response rate of 10%. In each camp, two men or women from two households participated in the survey aimed at measuring the level of knowledge of malaria and use of preventive methods. The survey was administered by three trained data collectors used to collect data for nomad immunization programmes. Each data collector was accompanied by a nomad translator although data collectors themselves understood the local language.

#### Qualitative data collection

Focus groups (FGD) with mothers were conducted to explore perceptions of malaria and knowledge of malaria control interventions. Topic guides were based on those of the National Malaria Control Programme (PNLP) Senegal, and by Moore et al. [39, 40]. The FGDs were carried out in six nomadic camps divided into three FGDs including Arabs (two camps in Dourbali), Fulani (two camps in Korbol) and Dazagada (two camps in Massaguet). The camps were selected in collaboration with nomadic leaders of each group and according to logistical convenience, including geographic accessibility. FGD participants were women (mothers) over 18 years old who were selected by women of the camp. Each FGD included eight mothers (four women from two camps); to obtain a variety of views older and younger women were included. FGDs were held in a calm and neutral space, usually an isolated hut in the home of a camp leader and carried out in local languages used by participants including Arabic, Fulani and Dazagada. One research assistant facilitated the session by leading the discussion using a topic guide, while the other research assistant managed the digital recorder and took detailed notes.

A semi-structured interviews were conducted with community leaders to explore the link between the nomadic lifestyle and the risk of malaria. Leaders of all six camps were invited to participate in the interviews; all agreed to participate. Interviews were conducted by the same research assistants, and were held in a calm and neutral space in an isolated tent in the home of a camp leader and audio-recorded with participant consent.

### Data analysis

Survey data assessing knowledge of malaria and the use of preventive interventions was analysed using Chi-squared test and analysis of variance (ANOVA). The Chi-squared test was used to assess the relationship between categorical variables and nomadic groups, including gender (female/male) and marital status (divorced/monogamous/polygamous/widowed). The ANOVA was used to assess the differences between means of age, household size and number of children under 5 years by nomadic groups (Arab, Daza and Fulani). The socio-demographic characteristics of the interviewees were presented and an analysis of respondents’ knowledge including that related to period of high frequency of malaria (June/July–September/October), group most at risk of malaria (children under 5 years/Pregnant women), cause of malaria (mosquito, water/heat/sun, hunger/food, destiny/fate/act of God/mystic), symptom or sign of malaria (fever/chills/muscle pain/stomach pain/diarrhoea/nausea/vomiting), preventive method known (LLIN/IPT/SMC/traditional), moment to go under net (before/after 7PM), target group for IPT, target group for SMC, knowledge of number of SMC cycles and its posology, practices (sleeping under mosquito net), perceptions and attitudes towards malaria, use of prevention methods (ownership of mosquito net, mosquito net installed, night spend under mosquito net, household visited by SMC distributor, women received ITP dose during last pregnancy), and health care-seeking behaviour (first intention for service delivery method, preference for provider, reason for choice of provider) using Stata 13.

After each interview and discussion, there was a debriefing session with the two research assistants in order to adapt the topic guide when necessary to improve data collection. Interviews and discussions were transcribed verbatim from the local language into French by the research assistants (WD and MDR). The transcribed data were read twice and the research assistants also listened repeatedly to the audio material in order to improve the quality of transcription. Then transcribed data were checked by another author (BH) fluent in the three local languages and French. A coding frame, and preliminary themes were identified by BH. Preliminary themes were discussed by BH, AM and WD and reviewed by HS and AM. The whole team agreed the final themes.

## Results

### Survey results

Between 2 February and 28 March 2021, 78 households spread over 40 nomad camps in Dourbali (n = 11 Arab camps, n = 6 Fulani camps), Massenya (n = 6 Arab camps) and Massaguet (n = 17 Dazagada camps) were surveyed. Most interviewees were female (70.5%) and married (97.4%). Respondents’ mean age was 38.1 years (95% CI 36.7–39.5) and there were significant differences between nomadic groups, with respondents from Daza group being older. The mean household size was 8.4 (95% CI 7.7–9.0), with no significant difference between nomadic groups. Households surveyed had an average of 2.5 children under 5 years (95% CI 2.1–2.9) (Table 1). Proportion of respondents by marital status varied significantly by nomadic groups with monogamy more represented in Arab and Daza groups whereas polygamy more represented in Fulani group.

**Table 1:** Socio-demographic characteristics of the survey participants, frequency (%)

Variable	Category	Arab (n = 33)	Daza (n = 34)	Fulani (n = 11)	Statistic	P-value (for difference between groups)	All (N = 78)
Gender	Female	27 (81.8)	20 (58.8)	8 (72.7)	Chi square = 4.3	0.12	55 (70.5)
	Male	6 (18.2)	14 (41.2)	3 (27.3)			23 (29.5)
Marital status	Divorced	0 (0.0)	1 (2.9)	0 (0.0)	Chi square = 12.3	0.06	1 (1.3)
	Monogamous	17 (51.5)	28 (82.4)	5 (45.5)			50 (64.1)
	Polygamy	15 (45.5)	5 (14.7)	6 (54.5)			26 (33.3)
	Widowed	1 (3.0)	0 (0.0)	0 (0.0)			1 (1.3)
Age	Mean	33.1	44.3	33.8	F = 15.4	0.00	38.1
	95% CI	(31.1–35.1)	(42.1–46.6)	(30.4–37.4)			(36.7–39.5)
Household size	Mean	9.5	7.5	7.8	F = 2.8	0.85	8.4
	95% CI	(8.4–10.6)	(6.6–8.5)	(6.3–9.7)			(7.7–9.0)
Number of children under 5 years	Mean	2.4	2.7	2.3	F = 0.8	0.43	2.5
	95% CI	(1.9–3.0)	(2.1–3.3)	(1.5–3.5)			(2.1–2.9)

CI = 95% Confidence Interval

### Knowledge of malaria

All respondents (78) had ever heard of malaria and the majority believed malaria is more frequent in the rainy season (80.8% reporting increased number of cases in July–September, and 38.5% in June and October). The knowledge of the period of high incidence of cases of malaria varies significantly by nomad groups; respondents from Arab and Fulani groups frequently mentioned the rainy season while those from Daza groups did less often. Regarding groups vulnerable to malaria, respondents most frequently mentioned children under 5 years, pregnant women and adults (84.6%, 64.1% and 74.4%), with those mentioning children under 5 years and adults varying significantly by nomadic groups (Table 2).

**Table 2:** Knowledge of respondents regarding malaria transmission

Variable	Category	Arab (n = 33)	Daza (n = 34)	Fulani (n = 11)	Chi square Statistic	P-value	All (N = 78)
Period of high frequency malaria	June	22 (66.7)	2 (5.9)	6 (54.5)	27.5	0.00	30 (38.5)
	July–September	31 (93.9)	22 (64.7)	10 (90.9)	10.1	0.01	63 (80.8)
	October	21 (63.6)	3 (8.8)	6 (54.5)	22.7	0.00	30 (38.5)
Group most at risk of malaria	Children under 5 years	33 (100.0)	22 (64.7)	11 (100.0)	18.4	0.00	66 (84.6)
	Pregnant Women	18 (54.5)	22 (64.7)	10 (90.9)	4.8	0.09	50 (64.1)
	Adult	29 (87.9)	18 (52.9)	11 (100.0)	15.1	0.00	58 (74.4)
	Disabled	5 (15.2)	4 (11.8)	4 (36.4)	3.7	0.16	13 (16.7)
Vector cause of malaria	Mosquito	21 (63.6)	15 (44.1)	3 (27.3)	5.2	0.07	39 (50.0)
Environmental cause of malaria	Water	28 (84.8)	19 (55.9)	10 (90.9)	9.2	0.01	57 (73.1)
	Heat/Sun	15 (45.5)	17 (50.0)	7 (63.6)	1.1	0.58	39 (50.0)
Poor nourishment cause of malaria	Hunger	2 (6.1)	12 (35.3)	1 (9.1)	10.1	0.01	15 (19.2)
	Food	19 (57.6)	9 (26.5)	6 (54.5)	7.2	0.03	34 (43.6)
Religious/supernatural forces cause of malaria	Destiny/fate/act of God	1 (3.0)	0 (0.0)	1 (9.1)	2.8	0.25	2 (2.6)
	Mystic/witchcraft	3 (9.1)	2 (5.9)	2 (18.2)	1.5	0.46	7 (9.0)
Sign/symptom malaria	Fever	30 (90.9)	13 (38.2)	11 (100.0)	27.5	0.00	54 (69.2)
	Chills	14 (42.4)	3 (8.8)	3 (27.3)	9.9	0.01	20 (25.6)
	Muscle pain	26 (78.8)	32 (94.1)	10 (90.9)	3.7	0.16	68 (87.2)
	Stomach pain	9 (27.3)	10 (29.4)	5 (45.5)	1.3	0.51	24 (30.8)
	Diarrhoea	10 (30.3)	7 (20.6)	2 (18.2)	1.1	0.57	19 (24.4)
	Nausea	12 (36.4)	4 (11.8)	2 (18.2)	5.9	0.05	18 (23.1)
	Vomiting	30 (90.9)	2 (5.9)	7 (63.6)	49.4	0.00	39 (50.0)

Concerning the causes of malaria transmission, responses varied between nomad groups. Most mentioned mosquitoes (including 63.6% respondents from the Arab group), in addition to environmental causes of malaria such as rainfall (73.1%) and heat or sun (50%). Muscle pain, fever, and nausea or vomiting were the most frequently mentioned symptoms of malaria, reported by 87.2%, 69.2% and 73.1% of all respondents, respectively.

### Knowledge of malaria preventive methods

Most respondents believed malaria can be prevented. As preventive methods, LLIN, SMC, IPT, and traditional methods were mentioned by 88.5%, 23.1%, 17.9% and 10.3% of respondents, respectively. While mosquito nets were the most widely-known method, 94.9% of respondents reported using a mosquito net recently after 7 PM, with respondents from Arab and Daza groups more likely to report this method than respondents from Fulani groups. Most respondents from Fulani group (54.5%) mentioned IPT among preventive methods; almost none of the respondents across the three groups correctly identified the target group for IPT, however. Concerning SMC, 55.1% of respondents identified children under 5 years as the target of SMC but just 11.5% and 15.6% of respondent were able to mention the correct number of SMC cycles and the correct dosing of SMC medicines, respectively (Table 3).

**Table 3:** Knowledge and attitudes of respondents regarding malaria preventive methods

Variables	Category	Arab (n = 33)	Daza (n = 34)	Fulani (n = 11)	Chi2 Statistic	P-value
Preventive methods mentioned	LLIN	29 (87.9)	29 (85.3)	11 (100.0)	1.8	0.41
	IPT	8 (24.2)	0 (0.0)	6 (54.5)	18.3	0.00
	SMC	10 (30.3)	1 (2.9)	7 (63.6)	4.4	0.11
	Traditional	3 (9.1)	2 (5.9)	3 (27.3)	4.2	0.12
When to go under mosquito net	≥ 7 pm	31 (93.9)	34 (100.0)	9 (81.8)	5.7	0.06
Group IPT	Adult	4 (12.1)	4 (11.8)	0 (0.0)	36.8	0.00
	U5	0 (0.0)	1 (2.9)	3 (27.3)		
	Pregnant women	0 (0.0)	11 (32.4)	0 (0.0)		
	Young	0 (0.0)	3 (8.8)	0 (0.0)		
	Every body	29 (87.9)	15 (44.1)	8 (72.7)		
Group SMC	Adult	3 (9.1)	0 (0.0)	0 (0.0)	8.0	0.02
	U5	10 (30.3)	24 (70.6)	9 (81.8)		
	Pregnant women	0 (0.0)	1 (2.9)	0 (0.0)		
	Young	0 (0.0)	7 (20.6)	0 (0.0)		
	Every body	20 (60.6)	2 (5.9)	2 (18.2)		
Know the number of SMC cycles		6 (18.2)	1 (2.9)	2 (18.2)	4.4	0.11
Know the SMC posology		9 (28.1)	1 (2.9)	2 (18.2)	8.0	0.02

*U5*  Children under 5 years old

### Reported use of malaria preventive methods

Mosquito nets were the most widely-reported preventive method used among participants. Nearly 75% reported owning at least one mosquito net, and 42.3% reported owning at least one LLIN. Also, nearly 60% of respondents declared they had slept under mosquito net the last night previous to survey. Mosquito net ownership and usage varied between nomad groups; the Daza group had the lowest proportion who reported owning (41.2%) and using (5.9%) a mosquito net. LLINs were followed by SMC as the next-most common method; 33.3% of households received a visit by SMC community distributors in 2020. Coverage of SMC and IPT treatment depended on the clinic catchment area where nomad groups reside. The coverage of SMC and IPT were higher among Arab and Fulani groups surveyed in Dourbali and Massenya districts than Daza groups surveyed in Massaguet district. Respondents from Fulani communities reported the highest percentage of household visits by SMC distributors. Of all mothers interviewed, just 12.8% said they received at least one IPT dose during their last pregnancy. This percentage was particularly low in Dazagada communities (see Table 4).

**Table 4:** Coverage of malaria preventive methods

Variables	Arab (n = 33)	Daza (n = 34)	Fulani (n = 11)	Chi2 statistic	P-value
Own at least one mosquito net	33 (100.0)	14 (41.2)	11 (100.0)	34.8	0.00
Own at least one LLIN	26 (78.8)	1 (2.9)	6 (54.5)	40.3	0.00
Mosquito net installed	3 (9.1)	0 (0.0)	1 (9.1)	3.3	0.20
Last night slept under mosquito net	32 (100.0)	2 (5.9)	11 (100.0)	69.2	0.00
Household visit by SMC distributor	16 (48.5)	1 (2.9)	9 (81.8)	29.2	0.00
Received at least one dose IPT during last pregnancy*	6 (22.2)	0 (0.0)	3 (37.5)	7.2	0.03

^*^Arab (n = 27), Daza (n = 20) and Fulani (n = 8)

**Table 5:** Treatment seeking behaviour

Variables	Category	Arab (n = 33)	Daza (n = 34)	Fulani (n = 11)	Chi2 statistic	P-value	All (N = 78)
First intention for service delivery method	Health facilities	25 (75.8)	0 (0.0)	7 (63.6)	50.3	0.00	32 (41.0)
	Traditional drug	3 (9.1)	1 (2.9)	1 (9.1)			5 (6.4)
	Informal drug sellers	4 (12.1)	32 (94.1)	3 (27.3)			39 (50.0)
	Other	1 (3.0)	1 (2.9)	0 (0.0)			2 (2.6)
Preferred health service provider	Nurse/midwife	12 (36.4)	0 (0.0)	4 (36.4)	53.7	0.00	16 (20.5)
	Marabout	3 (9.1)	0 (0.0)	0 (0.0)			3 (3.9)
	Physician	13 (39.4)	0 (0.0)	4 (36.4)			17 (21.8)
	Local drug seller	5 (15.2)	34 (100.0)	3 (27.3)			42 (53.8)
Reason for choice of the clinician	Cost of health care	13 (39.4)	34 (100.0)	6 (54.5)	31.1	0.00	53 (68.0)
	Severity of sickness	16 (48.5)	0 (0.0)	5 (45.5)			21 (26.9)
	Support	4 (12.1)	0 (0.0)	0 (0.0)			4 (5.1)
Opinion of quality of service	Good	23 (69.7)	23 (67.6)	6 (54.5)	22.7	0.00	52 (66.7)
	Bad	2 (6.1)	10 (29.4)	4 (36.4)			16 (20.5)
	Very good	8 (24.2)	1 (2.9)	1 (9.1)			10 (12.8)
Person responsible for decision on treatment	Other member	1 (3.2)	0 (0.0)	0 (0.0)	12.4	0.02	1 (1.3)
	Head of household	28 (90.3)	15 (44.1)	10 (90.9)			53 (69.7)
	Female head of household	2 (6.5)	19 (55.9)	1 (9.1)			22 (29.0)

### Treatment-seeking behaviour

When asked about their first recourse for malaria treatment, 41% and 50% of respondents mentioned health facilities and informal drug sellers respectively. Preferences for service delivery method varied between nomadic groups. Informal drug sellers were the most frequently-reported malaria treatment options among Daza respondents (94.1% of respondents). Regarding preferred health service provider for malaria treatment, 20.5% of respondents mentioned a preference for a nurse or midwife and 53.8% of respondents mentioned local drug seller; this preference varied between nomadic groups. Cost of health care and the severity of sickness were the main reasons for the choice of health service provider; these reasons were mentioned by 67.9% and 26.9% of respondents respectively. In addition, 78.5% of respondents expressed a positive opinion of services provided by these sources mentioned. Meanwhile, the household member responsible for decision-making on malaria treatment was most commonly a male head of household, with the exception of the Daza, among whom 55.9% of respondents mentioned a female head of household (See Table 5).

### Mothers’ and camp leaders’ perceptions of malaria and control interventions

In all three FGDs most women were aged over 30 years and had no formal education: 41% of mothers had no education and 32% had a Koranic education. More than half of the mothers had at least one child under 5 years (55%), and just over a third had their last pregnancy less than 1 year prior (36%). Only four women out of 22 had attended ANC during their last pregnancy (Table 6). Interviews were held with leaders of each of the six camps visited for FGD; all were male and aged over 30 years.

**Table 6:** Socio-demographic characteristics of mother who participated in each FGD

Variables	Category	Arab (n = 6)	Daza (n = 8)	Fulani (n = 8)
Age (years)	20–29	0	0	2
	30–49	6	8	6
Education	None	6	2	1
	Koranic	0	0	7
	NA	0	6	0
Number of children	3–4	1	0	2
	> 5	5	4	6
	NA	0	4	0
Number of children under 5 years	0	0	2	4
	1–2	6	2	4
	NA	0	4	0
Duration from last pregnancy (years)	0–1	3	0	5
	> 1	3	4	3
	NA	0	4	0
ANC during last pregnancy	Yes	0	1	3
	No	6	3	5
	NA	0	4	0

*NA *  no data available

Four overarching themes around the social representation of malaria and malaria risk, perspectives on malaria prevention and malaria treatment practices among nomadic groups were identified (Additional File 1: Table S7).

### Social representation of malaria risk

#### Perception of malaria risk among nomadic groups

Nomadic leaders and mothers expressed consistent views on the risk of malaria among their groups. Firstly, they were certain that malaria is a disease that “attacks everyone”, from young to old and men and women. They used the expression ‘big and small’ affected by malaria to imply that the disease affects everyone without exception. Mothers in all three discussions and all the leaders interviewed mentioned that malaria was the biggest health challenge in their community.

Lastly, both leaders and mothers explained how their nomadic lifestyle put them in contact with mosquitoes and, therefore, at risk of malaria. For example, the leaders reported that “*flies and mosquitoes… hunt north*”, which implies that when mosquito density is high in their temporary settlement in the south this compels nomads to move to the north of the country; it is an obligation to travel. Leaders also made a connection between the nomads’ temporary huts, often adjacent to their animal compounds, which are usually dark enclosed spaces. They believed that because mosquitoes are attracted to dark places and the heat and odor of animals, this put them at risk of mosquito bites and therefore malaria. The quotes below illustrate the social representation of risk; see Additional File 2: additional quotes in Box S1.1.

“We think malaria attacks everyone big and small. Malaria bothers everyone” (FGD Arab Dourbali)

“In the rainy season, flies and mosquitoes drive us for the north” (KII Daza Massaguet 2)

“We move with these diseases [malaria]. They abuse during all times of the year”. (KII Arab Dourbali).

### Social representation of malaria

#### Diverse local explanations for cause of malaria

Most of the nomadic women participating in FGDs and the leaders interviewed were aware of established biomedical causes of malaria; for example, the mothers associated malaria with mosquitoes and leaders linked it to the rainy season. However, a plethora of other causes were mentioned; some believed that malaria was due to nomads’ living conditions, including poor nourishment, hunger and food insecurity. Others mentioned physical exertion due to the nomadic lifestyle that involved long walks during transhumance; this was particularly the case for Arab and Daza groups and less for Fulani in the process of adopting a sedentary lifestyle. Others attributed the cause of malaria to the physical environment such as the presence of moisture or rainwater, cold or plenty of sun. The cold was more often mentioned by the Fulani group, who also describe using blankets as prevention against malaria. Leaders indicated “… cool water in the rainy season”, and “the humidity” brings malaria. Contrary to the leaders, mothers believed malaria is caused by heat or ‘abundant sun’. Mothers from Fulani and Arab groups also referred to religious causes such as acts of God.

“It is God who makes malaria rife [Laughs]” FGD Fulani Korbol

“Malaria is permanent during the rainy season. So it is the humidity that brings malaria” KII Fulani Korbol

“We are in the sun, we have no food. If we were in the shade, we are not going to suffer from malaria. Also, there are mosquitoes too” FGD Arab Dourbali

“We go south where there is water, the sun is also abundant, we are tired, this is what gives disease” FGD Daza Massaguet

“There are also mosquitoes which cause malaria despite the fact that we sleep under mosquito nets” FGD Fulani Korbol.

#### Manifestation and symptoms of malaria are well known

While some nomads generally knew the biomedical causes of malaria and its manifestations and symptoms, some also mentioned local explanations. In all three nomadic groups, anorexia (refusal of food), fever, chills, stomach aches and nausea were commonly described as manifestations and symptoms of malaria.

In addition to describing malaria manifestations and symptoms, mothers also described malaria fever in great detail. For example, women mentioned that fever is intermittent, ‘accompanied by the refusal of breast milk’ and that fever can also be ‘followed by a cold’. Mothers were quite specific in describing a pattern of feeling well in the morning followed by’suffering’ in the evening and recognized the ‘rhythm of a sick day and a healthy day’ as malaria. These words effectively designate discontinuous recurring fevers as malaria.

“In the morning, she is better, however in the evening, she goes to bed due to the pain. It is in this rhythm of a sick day and another healthy day that we say it is malaria. Also, a persistent cold reaches the sick person in the chest. Even being in hospital, this cold persists” FGD Fulani Korbol.

“The body hurts, the head also hurts, the person has the chills and she also vomits” FGD Daza Massaguet

“Malaria that tires people out. It creates stomach aches, headaches. It can kill too” KII Arab Dourbali

“The fever that makes one think of malaria is that which is accompanied by the refusal of breast milk. When the child refuses to breastfeed” FGD Arab Dourbali.

### Perspectives on malaria prevention

#### Knowledge of intermittent preventive treatment (IPT) varied among nomadic women

Many nomadic women seemed unaware of IPT for preventing malaria in pregnancy. Several claimed they had ‘not heard of it’. However, others who claimed they had ‘heard about it’ described following a nurse or doctor’s direction in relation to antenatal care and IPT. The few participants who were familiar with IPT said that it protects pregnant women and their children against malaria by reducing the frequency of episodes of the disease.

“We have not heard of it and no one has provided such treatment” FGD Arab Dourbali

“I respect the appointment of the month that the nurse gives me during ANC [antenatal care] and IPT. What the nurse says there for the pregnant woman to do” FGD Fulani Korbol

“The advantage of these drugs is that they protect pregnant women and their children against malaria” FGD Fulani Korbol

“This is to reduce the frequency of illness” FGD Daza Massaguet

#### Seasonal Malaria Chemoprevention (SMC) is not commonly known or used by nomadic groups

Nomadic women did not seem to be familiar with SMC; many said they ‘had not heard’ of SMC. Some women from Arab and Daza groups who had, explained that even when their nomadic group was present in an SMC-eligible district, their camps were excluded and did not receive visits from community distributor to administer the SMC doses. Nomadic leaders from Dourbali said even when health facilities are aware of their presence in the catchment area “they didn’t come to us”, which seemed to indicate a feeling of exclusion or discrimination against nomadic groups.

“We have not heard of this drug. We did not hear anything” FGD Arab Dourbali

“We have not heard of it except the mosquito net that we heard about. Perhaps by going to the doctor [clinician] in Massaguet we will obtain” FGD Daza Massaguet

“No! They don't come. We heard about it, but they didn't come to us” KII Arab Dourbali

#### Insecticide treated nets widely known but not freely available

The mosquito net appeared to be the most popular and widely-known malaria prevention method among all three nomadic groups. However, women in all three groups mentioned that they had too few nets for everyone in their household, or that they were not in good condition, for example several mentioned that ‘they are torn’. Except for nomads adopting a sedentary lifestyle (the Fulani group in Korbol), women’s reports suggest that insecticide treated nets are not provided for free and that they have to buy them from the market. The leaders emphasized how their nomadic groups feel excluded from health interventions, as one Daza leader expressed “why is the state just watching us but not stepping in to help us”. However, the discussions with mothers suggested that it is common for nomads to renew the mosquito nets at the start of the rainy season. All participants stated a preference for spacious mosquito nets impregnated with insecticide.

“Sometimes, the mosquito net is not sufficient. The husband and his wife are fighting over the mosquito net. Find us mosquito nets. We get it with our own money” FGD Arab Dourbali

“We heard about it but we didn’t receive it. Nomads have nothing. We pay for our mosquito nets, our medicines and our food.” KII Arab Dourbali

“We buy it and several of us sleep in it, it rips apart. Anyway, at the start of the rainy season we buy more. […]. Two people can sleep there, a mother and her children can sleep there as well as a father” FGD Daza Massaguet.

“LLINs are very large and several people can sleep in a single net. In addition, mosquitoes cannot get in because there are products that drive them away” FGD Fulani Korbol.

#### Various uses for mosquito nets

Most nomadic women recognized that mosquito nets are intended for use by children and pregnant women for protection from mosquito bites, and that use is recommended during the rainy season. However, several women mentioned various uses for mosquito nets including protection from other insect bites. Some Fulani women indicated that the nets ‘do not protect us [children and mother] against malaria’ and requested blankets instead.

“First, it is the children who have to sleep under a mosquito net. Adults can protect themselves in other ways” FGD Arab Dourbali

“Mosquito nets are primarily intended for our children but also for pregnant women” FGD Fulani Korbol

“During the mosquito season, as soon as it gets dark, we use it. We use the mosquito net during the rainy season when there are mosquitoes” FGD Daza Massaguet

“We use the mosquito net against mosquitoes also bees whose bite hurts. One of the obligations to use the mosquito net” FGD Arab Dourbali

“The mosquito nets we are given protect our children and ourselves against mosquitoes. But, they do not protect us against malaria. Bring us blankets” FGD Fulani Korbol.

### Malaria treatment practices in nomadic households

#### Treatment strategies for malaria are driven by financial considerations

Three remedies for treatment were mentioned by the nomads: modern medicine, traditional medicine and street drugs. Health facilities were often mentioned despite complaints about financial barriers to access. The Arab groups particularly mentioned that ‘without money, no one will take care of us’. On the contrary, the Fulani who live a more sedentary lifestyle have the possibility of being treated on credit at public health centre.

“We go to the nearest hospital to us, either N’Djamena, Dagana or Bachom, we take the vehicle to go there” FGD Daza Massaguet

“Even when we don't have the money, we go to the hospital for treatment on credit” FGD Fulani Korbol

“We go to hospitals. The latter also do not process it for free. Without money, nobody will take care of us” FGD Arab Dourbali.

#### The head of household as the key decision maker for curative treatments and prevention methods

The head of household is typically responsible for decision-making for malaria treatments or the purchase of mosquito nets. In addition, male heads of household are usually responsible for providing the necessary funds to pay for prevention methods and treatment. In the absence of a male head of household, responsibility typically falls on the camp leader. As far as it concerns mothers, they are responsible to ‘take care of … child’ by giving drugs or using nets and sometimes helping their husband financially.

“It was the husband who bought the mosquito net” FGD Arab Dourbali

“It is the child's father who is involved in the care decision, in case of his absence, it is the camp leader. It is the mother who should take care of it when it comes to a child” FGD Daza Massaguet.

“The father of the child has to bring the child to the hospital and pay the bills; (Laughs) It's the man who takes charge when the child gets sick.” FGD Fulani Korbol.

#### Local treatment practice against malaria

Nomad women and leaders mentioned hospitals as a source of treatment. However, recourse to traditional medicine, street drug sellers and self-medication are also quite common for Arab and Daza groups. Both leaders and women claimed to use products found in their environment such as ‘koulkul tree leaves’, ‘camel urine’ and ‘beef urine’ or ‘milk butter’; this is particularly highlighted in Arab and Daza groups, while the Fulani group of Korbol, who are in the process of shifting to a more sedentary lifestyle, mentioned visiting the health centre. To manage the symptoms of malaria, Daza women ‘soak some cloth in water and put on the child’ to reduce fever and ‘cover him’ in case of shivering.

“We go to the seller along the street and also to the well-built hospitals” FGD Arab Dourbali

“We soak some cloth in water and we put on the child. In case of shivering, we cover him, if we have tablets such as paracetamol and nivaquine we also give it” FGD Daza Massaguet

“We have no other recourse other than hospitals. Otherwise, we also use "koulkoul" tree leaves and camel urine” KII Arab Dourbali.

## Discussion

This study improves understanding of knowledge and awareness of malaria and perceptions of malaria control interventions among the main nomadic groups in Chad. The results show that nomads are aware of the risk of malaria and know about its causes and symptoms, but cultural-specific interpretations of the disease coexist. Reported use of preventive interventions such as SMC and IPT is very low and few women are aware of them. The use of LLIN is still not widespread because net distribution campaigns often do not reach nomad camps. In case of a malaria episode, mothers seek clinicians, informal drug sellers in the street or market for self-medication, or traditional medicine depending on access to financial resources. Interviews with nomad leaders and discussions with women provide insights into: (i) the social representation of malaria risk, (ii) the social representation of malaria, (iii) perspectives on malaria prevention and (iv) malaria treatment practices.

The study revealed that there is a social representation of malaria risk. This representation drives self-adaptation of nomads and influences their lifestyle. For example, nomads mitigate risk by moving to the north of the country. However, while moving to the north they may transmit malaria to areas where the settled population has not yet recorded any cases as found elsewhere [10] and when they leave north areas they can be vulnerable to malaria upon entry into a high-transmission zone [7, 13]. Thus, the WHO guidelines for malaria [41] recommend that nomads and migrants should receive chemoprophylaxis to prevent malaria while moving permanently or temporarily within or outside their usual place of residence. Chemoprophylaxis can suppress the blood stage of malaria infections, thereby preventing malaria episodes. However, in Chad there is a need to adapt and implement the WHO recommendations specifically for nomadic groups, probably via pilot studies to test out different approaches and ways of delivering prophylaxis to highly mobile populations.

Another key theme, the social representation of malaria, comprised local explanations of the cause of the disease as found elsewhere [42–45] and knowledge of its manifestation and symptoms. Local explanation of malaria causes were mostly linked to the environment and lifestyle of nomads. For example, some reported a belief that malaria is caused by malnourishment; this could be explained by the association between malnutrition and anaemia which, in turn predisposes malnourished individuals to malaria. Moreover, the chronic malnutrition among under-fives in Chad was 30.5% in 2020 [46], and malnutrition is more prevalent among nomadic populations than their sedentary counterparts [6]. Other participants attributed malaria to the physical environment, such as the presence of moisture, cold or high temperatures. In some local languages of Chad malaria is called “cold weather”; this may be due to its association with cold and cloudy weather, which represent conditions during the high transmission season as mentioned elsewhere [42]. In addition, this social definition of malaria as “cold weather” can be explained also by the symptom that is “the chills”. Despite this, participants’ perceptions of the cause of malaria is oriented to the prevention mechanism they choose, with some participants asking for blankets to prevent malaria. These findings could be used to inform more appropriate, tailored health education to address misconceptions about the cause of malaria among nomadic communities.

On the side of perspectives on malaria prevention, the reported coverage of preventive interventions such as SMC is very low despite relatively high awareness, leading to feelings of exclusion. Nomadic children tend to miss SMC distributions because they are not included in census and health facilities do not count them in their catchment area [27], or health facilities are unaware of their temporary presence in their area. As for SMC, self-reports of IPT use were found to be very low (12.8%) and women had low awareness: this is consistent with other studies [1, 47, 48], and can be attributed to the fact that only 38.8% of mothers in rural areas of Chad attend antenatal care (ANC) [1]. The percentage of pregnant women who receive at least one dose of IPT stands at around 20% nationally [1, 47]. To tackle the low coverage of SMC and the low attendance of pregnant women to ANC (thus receiving IPT), integrating these malaria control methods to the nomad immunization programme of Chad could be a solution. In fact, this programme includes an independent system for tracking each nomad camp for immunization of children. The system gives the position of nomad camp and when the group is likely to be present in a health facility’ catchment area. Thus, if integrated, health centres in the Sahel region of Chad could prepare SMC and IPT medicines prior to arrival of nomad camps. To succeed in this integration, the national programme for health of nomad population (PNSN) and PNLP, under guidance of the Ministry of Health, should engage with the WHO to pilot this mechanism.

As far as perspectives on malaria prevention are concerned, mosquito nets are the most widely-known intervention with the highest coverage: around 75% of households in the study possessed at least one mosquito net (42.3% for LLIN). This high coverage has also been found in other studies in Chad which report coverage of 77.7% (67.1% for LLIN) [1] and 80% (73.4% for LLIN) [47]. However, nomads in the process of adopting a sedentary lifestyle have benefitted from mass distribution of LLINs on the contrary to the most mobile nomads which leads to a feeling of discrimination. Even when coverage of mosquito nets is high, the number of nets per household was not sufficient due to financial barriers as most nomads purchase nets themselves. To address this, firstly it is urgent for the PNLP to distribute LLINs to nomadic groups and secondly, for the next campaign, nomads should be integrated in the census and a team comprising members of nomadic community should be deployed to distribute LLINs [7, 26].

Regarding the use of mosquito nets, 58.4% of the survey respondents reported having slept under mosquito net the night before the survey, which is high considering the period of the study (dry season). Other studies in Chad have found also a comparative percentage of 60.1% [1], 37.6% [49] and 31.7% [47]. As found elsewhere [50, 51] high usage of nets can be explained by a perception that nets prevent insects bites rather than malaria. However, use of nets could be improved since only 5.1% of respondents acknowledged that they went under mosquito nets before 7 PM. Targeted health information for nomadic communities may be an important influence [7].

Financial considerations seem to drive malaria treatment practices among nomadic groups. For example, around half the nomads in the study mentioned informal drug sellers (50% of respondents) and health facilities (41% of respondents) as their first recourse for seeking malaria treatment. Other studies in Chad [47] found that 31.1% of respondents use informal drug sellers for obtaining treatment and advice. However, nomads are not facing the financial barriers in the same manner. In contrast with nomads in the process of adopting a sedentary lifestyle who can obtain treatment at health facilities on credit, those mostly practicing transhumance face even greater barriers for access to health. This situation has led nomads to turn to informal drug sellers in local markets or locally-available treatments such as tree leaves or animal urine. This situation must be tackled urgently to redress nomads’ perceptions of exclusion. Despite the fact that case management of simple malaria is free of charge in health facilities, this policy has yet to be fully applied in practice [52].

## Strengths and limitations

Survey data were based on self-reporting which could be influenced by nomads’ desire and demand for assistance from the government. In addition, malaria was reported using the local Arabic “*wired*”, which can also encompass symptoms such as fever, headache, and vomiting might as well have been due to other illnesses, resulting in misclassification.

Another challenge was the composition of the research team, which was primarily male. To minimize bias and ensure respondents could express themselves naturally, a nomadic translator was used although the research assistants could understand the local languages. In addition, for cultural reasons, and at the request of participants in one focus group, the use of a mat as a barrier to distance themselves from the research team was allowed. Working with camp women to select mothers to participate in FGDs may have led to selection bias, women may have recommended those likely to provide responses compatible with biomedical knowledge and ‘expected’ prevention and treatment-seeking behaviours. However, this is the first study to assess knowledge and explore the perception of malaria and preventive interventions among nomad communities in Chad.

## Conclusions

This study assessed knowledge and perceptions of malaria and preventive interventions among nomad communities. Nomads are aware of risk of malaria and their level of exposure increases due to their habitat and schedules for anti-malarial interventions should be adapted to seasonal patterns of movement. Malaria chemoprevention should be trialed as a pilot project to assess clinical and economic feasibility. Nomads often have culturally-specific interpretations of malaria and they are less aware of preventive interventions such as SMC and IPT. For that purpose, nomads’ knowledge of malaria and preventive interventions should be strengthened with tailored and culturally-appropriate health education. Regarding coverage of SMC and IPT, the Ministry of Health should lead a process to integrate these interventions with the existing nomad immunization programme led by the WHO. The thematic hierarchy identified in this study could helpfully inform future research on the health needs and barriers to accessing healthcare among nomadic groups, as well as malaria prevention strategies in mobile populations. Financial resources were a key barrier to mosquito net use and malaria treatment. In addition to integration of nomads into the national census, PNLP should enhance net distribution to nomads to reduce their risk of malaria and their perceptions of exclusion from health interventions. In addition, the current policy to provide free-of-charge care in health centres should be applied in practice without exclusion of nomads.

## Supplementary Information

**Additional file 1: Table S7:** Thematic hierarchy.

**Additional file 2:** **Box S1.1.** Social representation of malaria risk. **Box S1.2.** Social representation of malaria. **Box S1.3.** Perspectives on malaria prevention. **Box S1.4.** Malaria treatment practices in nomadic households.

**Additional file 3:** Form: Questionnaire_menage.

## Data Availability

The datasets generated during and/or analyzed during the current study are available on Mendeley public repository here. Qualitative data generated and/or analyzed during the current study can be found in Additional File 2.

## Abbreviations

ANC: Antenatal care
FGD: Focus Group Discussion
IPT: Intermittent Preventive Treatment
LLIN: Long-lasting insecticidal net
NMCP (PNLP): National Malaria Control Programme (*Programme National de Lutte contre le Paludisme*)
PNSN: National programme for health of nomad population
SMC: Seasonal Malaria Chemoprevention
UNICEF: United Nations Children's Fund
WHO: World Health Organization

## References

[1] Programme National de Lutte contre le Paludisme, INSEED. Rapport d’Enquête Nationale sur les indicateurs du paludisme 2017. Ndjamena: 2018. p. 165.

[2] Ministère de la Santé Publique. Annuaire de statistique sanitaire. Ndjamena; 2017. p. 183.

[3] Okeibunor JC, Onyeneho NG, Nwaorgu OC, I’Aronu N, Okoye I, Iremeka FU (2013). Prospects of using community directed intervention strategy in delivering health services among Fulani Nomads in Enugu State, Nigeria. Int J Equity Health.

[4] Seck MC, Thwing J, Fall FB, Gomis JF, Deme A, Ndiaye YD (2017). Malaria prevalence, prevention and treatment seeking practices among nomadic pastoralists in Northern Senegal. Malar J.

[5] Seck MC, Thwing J, Badiane AS, Rogier E, Fall FB, Ndiaye PI (2020). Analysis of anti-*Plasmodium* IgG profiles among Fulani nomadic pastoralists in northern Senegal to assess malaria exposure. Malar J.

[6] Bechir M, Schelling E, Hamit MA, Tanner M, Zinsstag J (2012). Parasitic infections, anemia and malnutrition among rural settled and mobile pastoralist mothers and their children in Chad. EcoHealth.

[7] Brieger W (2011). Malaria among nomads and migrants: a neglected people at risk. Afr Health.

[8] Moto DD, Daoud S, Tanner M, Zinsstag J, Schelling E (2004). Répartition de la morbidité dans trois communautés nomades du Chari-Baguirmi and Kanem, Chad. Med Trop (Mars).

[9] Schelling E, Daoud S, Daugla S, Diallo P, Tanner M, Zinsstag J (2005). Morbidity and nutrition patterns of three nomadic pastoralist communities of Chad. Acta Trop.

[10] Wiese M, Donnat M, Wyss K (2004). Utilisation d’un centre de santé par les pasteurs nomades arabes - une étude de cas au Kanem, Chad. Med Trop (Mars).

[11] Richard V (2004). Des dunes, des pierres et des coûts: approche de la santé nomades. Med Trop (Mars).

[12] Warsame M. Impact of population movement on malaria transmission in Somalia. In: Malaria and Development in Africa: a cross-sectoral approach. Washington DC: Am Assoc Adv Sci; 1991. p. 21.

[13] Zongo P. Modélisation mathématique de la dynamique de la transmission du paludisme. PhD Thesis, Université de Ouagadougou; 2009. https://tel.archives-ouvertes.fr/tel-00419519. Accessed 15 Jun 2020.

[14] OMS. Paludisme : projet de stratégie technique mondiale pour l’après-2015. Genève: Organisation Mondiale de la Santé; 2015.

[15] Plucinski MM, Chicuecue S, Macete E, Colborn J, Yoon SS, Kachur SP (2014). Evaluation of a universal coverage bed net distribution campaign in four districts in Sofala Province, Mozambique. Malar J.

[16] Lengeler C (2004). Insecticide-treated bed nets and curtains for preventing malaria. Cochrane Database Syst Rev.

[17] Gamble CL, Ekwaru JP, ter Kuile FO (2006). Insecticide-treated nets for preventing malaria in pregnancy. Cochrane Database Syst Rev.

[18] Sesay S, Milligan P, Touray E, Sowe M, Webb EL, Greenwood BM (2011). A trial of intermittent preventive treatment and home-based management of malaria in a rural area of The Gambia. Malar J.

[19] Bojang K, Akor F, Bittaye O, Conway D, Bottomley C, Milligan P (2010). A randomised trial to compare the safety, tolerability and efficacy of three drug combinations for intermittent preventive treatment in children. PLoS ONE.

[20] Chatio S, Ansah NA, Awuni DA, Oduro A, Ansah PO (2019). Community acceptability of seasonal malaria chemoprevention of morbidity and mortality in young children: a qualitative study in the Upper West Region of Ghana. PLoS ONE.

[21] Cissé B, Sokhna C, Boulanger D, Milet J, Bâ EH, Richardson K (2006). Seasonal intermittent preventive treatment with artesunate and sulfadoxine-pyrimethamine for prevention of malaria in Senegalese children: a randomised, placebo-controlled, double-blind trial. Lancet.

[22] Dicko A, Diallo AI, Tembine I, Dicko Y, Dara N, Sidibe Y (2011). Intermittent preventive treatment of malaria provides substantial protection against malaria in children already protected by an insecticide-treated bednet in Mali: a randomised, double-blind, placebo-controlled trial. PLoS Med.

[23] Druetz T, Corneau-Tremblay N, Millogo T, Kouanda S, Ly A, Bicaba A (2018). Impact evaluation of seasonal malaria chemoprevention under routine program implementation: a quasi-experimental study in Burkina Faso. Am J Trop Med Hyg.

[24] Konaté AT, Yaro JB, Ouédraogo AZ, Diarra A, Gansané A, Soulama I (2011). intermittent preventive treatment of malaria provides substantial protection against malaria in children already protected by an insecticide-treated bednet in Burkina Faso: a randomised, double-blind, placebo-controlled trial. PLoS Med.

[25] Tagbor H, Antwi GD, Acheampong PR, Bart Plange C, Chandramohan D, Cairns M (2016). Seasonal malaria chemoprevention in an area of extended seasonal transmission in Ashanti, Ghana: an individually randomised clinical trial. Trop Med Int Health.

[26] Sanogo Y. Accès des nomades des zones transfrontalières aux interventions sous directive communautaire ciblant le paludisme et les maladies tropicales négligées au Mali en 2018 [Internet]. Thesis, Université des Sciences, des Techniques et des Technologies de Bamako; 2020. https://www.bibliosante.ml/handle/123456789/3976. Accessed 25 Jul 2021.

[27] Azoukalné M, Laura D, Zana C, Djiddi Ali S, Charlotte W. Seasonal Malaria Chemoprevention (SMC) in Chad: understanding barriers to delivery of SMC and feasibility and acceptability of extending to 5–10 year old children in Massaguet district. Ndjamena: Malaria Consortium; 2021. p. 57.

[28] INSEED. Deuxième Récensement Général de la Population et de l’Habitat 2009. Ndjamena: Institut National de Statistique, des Etudes Economique et Démographique; 2012. p. 121.

[29] Ministère des Finances et du Budget. Rapport annuel. Ndjamena; 2018.

[30] Wiese M, Yosko I, Donnat M (2004). La cartographie participative en milieu nomade: un outil d’aide à la décision en Santé Publique - étude de cas chez les Dazagada du Barh-El-Gazal region in Chad. Med Trop (Mars).

[31] Ministère de la Justice, Chargé des Droits Humains. Rapport National d’Evaluation des vingt cinq (25) ans de mise en oeuvre de la déclaration et du programme d’action de Beijing Ndjamena; 2019. p. 81.

[32] CTA. Atlas d’élevage du Bassin du Lac Tchad. Wageningen. Pays-Bas; (CTA). 1996. p. 158.

[33] Clanet J. Géographie pastorale au Sahel central. Thèse d’Etat en Géographie humaine. Université Paris IV Sorbonne; 1994.

[34] Fuchs P (1996). Nomadic society, civil war, and the state in Chad. Nomadic Peoples.

[35] Wiese M. Health-vulnerability in a complex crisis situation. Implications for providing health care to nomadic people in Chad. Verlag für Entwicklungspolitik. Saarbrücken; 2004. p. 436.

[36] Mahamat A. Les logiques de la mobilité pastorales. Presentation in worshop presented at: Briefing des consultants sur la vaccination nomade. Ndjamena; 2019.

[37] Peto TJ, Tripura R, Sanann N, Adhikari B, Callery J, Droogleever M (2018). The feasibility and acceptability of mass drug administration for malaria in Cambodia: a mixed-methods study. Trans R Soc Trop Med Hyg.

[38] DataDyne Group LLC. Magpi. 2020.

[39] National Malaria Control Programme, Groupe SOTERCO. Enquete nationale sur les indicateurs du paludisme au Sénégal. Dakar; 2017. p. 191.

[40] Moore GF, Audrey S, Barker M, Bond L, Bonell C, Hardeman W (2015). Process evaluation of complex interventions: Medical Research Council guidance. BMJ.

[41] WHO. Guidelines for malaria. Geneva: World Health Organization; 2021. https://www.who.int/publications-detail-redirect/guidelines-for-malaria.

[42] Abate A, Degarege A, Erko B (2013). Community knowledge, attitude and practice about malaria in a low endemic setting of Shewa Robit Town, Northeastern Ethiopia. BMC Public Health.

[43] Deressa W, Hailemariam D, Ali A (2007). Economic costs of epidemic malaria to households in rural Ethiopia. Trop Med Int Health.

[44] Maslove DM, Mnyusiwalla A, Mills EJ, McGowan J, Attaran A, Wilson K (2009). Barriers to the effective treatment and prevention of malaria in Africa: a systematic review of qualitative studies. BMC Int Health Hum Rights.

[45] Fuge TG, Ayanto SY, Gurmamo FL (2015). Assessment of knowledge, attitude and practice about malaria and ITNs utilization among pregnant women in Shashogo District, Southern Ethiopia. Malar J.

[46] Direction de la Nutrition et des Technologies Alimentaires (DNTA). Rapport final de l’enquête nationale de nutrition 2020 réalisée par la méthodologie SMART. Ndjamena: Ministère de la Santé Publique et de la Solidarité Nationale; 2020. p. 68.

[47] INSEED, ICF International. Enquête Démographique et de Santé et à Indicateurs Multiples au Tchad (EDS-MICS) 2014–2015. Ndjamena: 2016. https://www.dhsprogram.com/pubs/pdf/FR317/FR317.pdf. Accessed 20 May 2020.

[48] Carlson M, Smith Paintain L, Bruce J, Webster J, Lines J (2011). Who attends antenatal care and expanded programme on immunization services in Chad, Mali and Niger? The implications for insecticide-treated net delivery. Malar J.

[49] Yandaï FH, Moundine K, Djoumbe E, Boulotigam K, Moukenet A, Kodindo ID (2016). Perception de risques du paludisme et utilisation des moustiquaires au Tchad. Int J Biol Chem Sci.

[50] Serengbe GB, Moyen J-M, Fioboy R, Beyam EN, Kango C, Bangue C (2015). Knowledge and perceptions about malaria in communities in four districts of the Central African Republic. BMC Res Notes.

[51] Adongo PB, Kirkwood B, Kendall C (2005). How local community knowledge about malaria affects insecticide-treated net use in northern Ghana. Trop Med Int Health.

[52] Azoukalné M, Avocksouma DA. La gratuité des soins de santé au Tchad - Évaluation et perspectives. L’Harmattan. Paris; 2016. https://www.editions-harmattan.fr/index.asp?navig=catalogue&obj=livre&no=51508. Accessed 16 May 2020.

