# Supplementary material for: Knowledge and social beliefs of malaria and prevention strategies among itinerant Nomadic Arabs, Fulanis and Dagazada groups in Chad: a mixed method study
Source: Malar J. 2022 Feb 19;21:56. doi: 10.1186/s12936-022-04074-0 (PMC8858476; doi:10.1186/s12936-022-04074-0)
Supplement: Supplementary file 1 — Additional file 1: Table S7: Thematic hierarchy. [file 12936_2022_4074_MOESM1_ESM.docx]

**Title: Knowledge and social beliefs of malaria and prevention strategies among itinerant Nomadic Arabs, Fulanis and Dagazada groups in Chad: a mixed method study**

**Authors :** Azoukalné Moukénet^1*^, Beackgoubé Honoré^2^, Helen Smith^3^, Kebfene Moundine^4^, Wang-Mbe Djonkamla^2^, Sol Richardson^5^, Makido Dormbaye^6^, Ngarkodje Ngarasta^7^, Ibrahima Seck^1^

# additional file 1

Table 7: Thematic hierarchy

| Overarching theme | Higher-order theme | Sub-theme |
| --- | --- | --- |
| 1. Social representation of malaria risk | Perception of malaria risk | Endemic status of malaria |
|  |  | Habitat increases risk of malaria |
|  |  | Mobility to the north of the country |
| 2. Social representation of malaria | Local explanation of malaria causes | Poor nourishment/ hunger/ food insecurity |
|  |  | Lifestyle (mobility) |
|  |  | Physical environment |
|  | Manifestation and symptoms of malaria | Weight loss, food refusal, fever, chills stomach etc |
| 3. Perspectives on malaria prevention | Awareness of IPT and SMC | Low awareness of SMC/ knowledge of IPT varied |
|  |  | Belief that nomads are excluded from campaigns |
|  |  | Reliance on clinician direction for IPT |
|  | Possession and use of LLINs | LLINs widely known and used |
|  |  | LLINs not freely available |
|  |  | Various uses reported |
| 4. Malaria treatment practices | Treatment choice driven by financial considerations | Health care is often not free for nomads |
|  |  | Treatment on credit |
|  | Decision maker for treatment and prevention | Head of household makes decisions about treatment and prevention |
|  | Local malaria treatment practices | Hospitals/ formal health facilities |
|  |  | Traditional medicine, street drug sellers and self-medication |
