# Supplementary material for: Knowledge and social beliefs of malaria and prevention strategies among itinerant Nomadic Arabs, Fulanis and Dagazada groups in Chad: a mixed method study
Source: Malar J. 2022 Feb 19;21:56. doi: 10.1186/s12936-022-04074-0 (PMC8858476; doi:10.1186/s12936-022-04074-0)
Supplement: Supplementary file 2 — Additional file 2: Box S1.1. Social representation of malaria risk. Box S1.2. Social representation of malaria. Box S1.3. Perspectives on malaria prevention. Box S1.4. Malaria treatment practices in nomadic households. [file 12936_2022_4074_MOESM2_ESM.docx]

# additional file 2

## Supplementary quotes

Box 1.1 Social representation of malaria risk

| **Perception of malaria risk**  *“We think malaria attacks everyone big and small. Malaria bothers everyone” (FGD Arab Dourbali)*  *“No one is spared from malaria, adults, children, pregnant women, everyone is affected” (FGD Daza Massaguet)*  *“In the rainy season, flies and mosquitoes drive us for the north” (KII Daza Massaguet 2)*  *“Malaria bothers us in the rainy season, in the dry season it is better” (KII Daza Massaguet 1)*  *“Malaria bothers us; in the rainy season and until now (dry season)” (FGD Daza Massaguet)*  *“We move with these diseases [malaria]. They abuse during all times of the year”. (KII Arab Dourbali)* |
| --- |

Box 1.2 Social representation of malaria

| **Local explanations for cause of malaria**  *“When we go to the hospital we are told it is malaria. We are told it is typhoid malaria, it is chronic malaria. They are the ones who know this. We don't know any of this. They are also the ones who say it is hunger. They recommend that we eat well” KII Arab Dourbali*  *“Malaria is suffering like any other. It is God who is the cause. That is not it!” FGD Arab Dourbali*  *“It is God who makes malaria rife [Laughs]” FGD Fulani Korbol*  *“It is God who is the cause” FGD Arab Dourbali*  *“For me, hunger causes malaria” FGD Fulani Korbol*  *“For me it is the presence of water and mosquitoes” FGD Daza Massaguet*  *“Since we got here, malaria has bothered children here a lot. Especially during the rainy season. And what causes malaria is caused by cold water especially during the rainy season” KII Fulani Korbol*  *“Malaria is permanent during the rainy season. So it is the humidity that brings malaria” KII Fulani Korbol*  *“We are in the sun, we have no food. If we were in the shade, we are not going to suffer from malaria. Also, there are mosquitoes too” FGD Arab Dourbali*  *“We are going in the South where there is water, the sun is also abundant, we are tired, that's what causes the disease” FGD Daza Massaguet*  *“We go south where there is water, the sun is also abundant, we are tired, this is what gives disease” FGD Daza Massaguet*  *“I think it is the cold that is causing malaria in our community. Then the cold gives the common cold. Our malaria is exactly chronic malaria. It does not leave the community” FGD Fulani Korbol*  *“And what causes malaria is caused by cold water especially during the rainy season. […] Malaria is permanent during the rainy season. So it’s the humidity that brings malaria.” KII Fulani Korbol*  *“There are also mosquitoes which cause malaria despite the fact that we sleep under mosquito nets” FGD Fulani Korbol*  *“For me it is the presence of water and the mosquitoes” FGD Daza Massaguet*  *“Also, there are mosquitoes too” FGD Arab Dourbali*  **Manifestation and symptoms of malaria**  *“The child will refuse breastfeeding” FGD Arab Dourbali*  *“[If the child can speak] The child will tell you that I have a headache, I have a fever, my chest hurts” FGD Arab Dourbali*  *“In the morning, she is better, however in the evening, she goes to bed due to the pain. It is in this rhythm of a sick day and another healthy day that we say it is malaria. Also, a persistent cold reaches the sick person in the chest. Even being in hospital, this cold persists” FGD Fulani Korbol*  *“The body hurts, the head also hurts, the person has the chills and she also vomits” FGD Daza Massaguet*  *“The child is crying, we touch the child's hand which is heating up, this is how we know it is malaria” FGD Daza Massaguet*  *“Malaria that tires people out. It creates stomach aches, headaches. It can kill too” KII Arab Dourbali*  *“The fever that makes one think of malaria is that which is accompanied by the refusal of breast milk. When the child refuses to breastfeed” FGD Arab Dourbali*  *“A fever followed by a cold. Nobody goes to sleep because of the crying; neither his mother nor his father” FGD Arab Dourbali* |
| --- |

Box 1.3 Perspectives on malaria prevention

| **Knowledge of Intermittent Preventive Treatment**  *“We have not heard of it and no one has provided such treatment” FGD Arab Dourbali*  *“I don't know” FGD Fulani Korbol*  *“I respect the appointment of the month that the nurse gives me during ANC [antenatal care] and IPT. What the nurse says there for the pregnant woman to do” FGD Fulani Korbol*  *“We have heard about it. It is the doctor [clinician] who gives. When the sun hits and we have no blood, when the mosquitoes bite, the doctor gives this product” FGD Daza Massaguet*  *“You only get this product when you travel to N’Djamena or Dagana or Massaguet. The doctor [clinician] determines the number of times” FGD Daza Massaguet*  *“The advantage of these drugs is that they protect pregnant women and their children against malaria” FGD Fulani Korbol*  *“This is to reduce the frequency of illness” FGD Daza Massaguet*  **Knowledge of Seasonal Malaria Chemoprevention**  *“We have not heard of this drug. We did not hear anything” FGD Arab Dourbali*  *“We have not heard of it except the mosquito net that we heard about. Perhaps by going to the doctor [clinician] in Massaguet we will obtain” FGD Daza Massaguet*  *“No! They don't come. We heard about it, but they didn't come to us” KII Arab Dourbali*  **Knowledge and availability of mosquito nets**  *“We know the mosquito net. Nothing else! We don't have houses. We are in the bush” FGD Arab Dourbali*  *“We only have one mosquito net. It is torn by the way” FGD Arab Dourbali*  *“Sometimes, the mosquito net is not sufficient. The husband and his wife are fighting over the mosquito net. Find us mosquito nets. We get it with our own money” FGD Arab Dourbali*  *“We always buy at the market. We haven't had it for free yet” FGD Arab Dourbali*  *“In our locality we are not distributed mosquito nets, there is no free mosquito net, we have to buy it” FGD Daza Massaguet*  *“We heard about it but we didn’t receive it. Nomads have nothing. We pay for our mosquito nets, our medicines and our food.” KII Arab Dourbali*  *“We buy it and several of us sleep in it, it rips apart. Anyway, at the start of the rainy season we buy more. [...]. Two people can sleep there, a mother and her children can sleep there as well as a father” FGD Daza Massaguet*  *“It is inconvenient when the mosquito net is small” FGD Arab Dourbali*  *“LLINs are very large and several people can sleep in a single net. In addition, mosquitoes cannot get in because there are products that drive them away” FGD Fulani Korbol*  *“State mosquito nets prevent mosquitoes from biting while some mosquitoes can get close and even enter them which is not of quality” FGD Daza Massaguet*  *“They don't have a water source, a hospital, or a shelter. […] Where we stop, we have no doctor, nor free government. All we have is with our own means” KII Arab Dourbali*  *“Otherwise we are the victim of injustice and we will want to expose this” FGD Arab Dourbali*  *“Why is the state just watching us but not stepping in to help us in recent years?” FGD Daza Massaguet*  **Uses of mosquito nets**  *“First, it is the children who have to sleep under a mosquito net. Adults can protect themselves in other ways” FGD Arab Dourbali*  *“Mosquito nets are primarily intended for our children but also for pregnant women” FGD Fulani Korbol*  *“During the mosquito season, as soon as it gets dark, we use it. We use the mosquito net during the rainy season when there are mosquitoes” FGD Daza Massaguet*  *“We use the mosquito net against mosquitoes also bees whose bite hurts. One of the obligations to use the mosquito net” FGD Arab Dourbali*  *“The mosquito nets we are given protect our children and ourselves against mosquitoes. But, they do not protect us against malaria. Bring us blankets” FGD Fulani Korbol*  *“In terms of prevention, we tie up the mosquito net which prevents mosquitoes from biting. [...]” FGD Daza Massaguet* |
| --- |

Box 1.4 Malaria treatment practices in nomadic households

| **Treatment strategies for malaria are driven by financial access**  *“The only thing is to go to the hospital to see the doctor” FGD Fulani Korbol*  *“We go to the nearest hospital to us, either N’Djamena, Dagana or Bachom, we take the vehicle to go there” FGD Daza Massaguet*  *“Even when we don't have the money, we go to the hospital for treatment on credit” FGD Fulani Korbol*  *“We go to hospitals. The latter also do not process it for free. Without money, nobody will take care of us” FGD Arab Dourbali*  *“Being in the hospital with his sick wife, he has to pay for the treatment. No one will take care of his wife when he has nothing in his pocket. If he knows someone in the hospital maybe.” KII Arab Dourbali*  *“Nomads have nothing. We pay for our mosquito nets, our medicines and our food.” KII Arab Dourbali*  **The head of household as key decision maker**  *“It was the husband who bought the mosquito net” FGD Arab Dourbali*  *“It is the child's father who is involved in the care decision, in case of his absence, it is the camp leader. It is the mother who should take care of it when it comes to a child” FGD Daza Massaguet*  *“The husband takes care of it financially. If he does not have the means, his wife can get involved financially” FGD Arab Dourbali*  *“When the husband is there he helps if necessary it is the camp leader. The doctor indicates the dosage, once the doctor has indicated, they even take it” FGD Daza Massaguet*  *“The father of the child has to bring the child to the hospital and pay the bills; (Laughs) It's the man who takes charge when the child gets sick.” FGD Fulani Korbol*  **Local treatment practice against malaria**  *“We go to the seller along the street and also to the well-built hospitals” FGD Arab Dourbali*  *“We soak some cloth in water and we put on the child. In case of shivering, we cover him, if we have tablets such as paracetamol and nivaquine we also give it” FGD Daza Massaguet*  *“We also take quinine [tablets]” FGD Fulani Korbol*  *“We have no other recourse other than hospitals. Otherwise, we also use "koulkoul" tree leaves and camel urine” KII Arab Dourbali*  *“For treatment you have to take the child to the doctor [clinician]; apart from the doctor, he must be given milk butter, beef urine” FGD Daza Massaguet* |
| --- |
