# Supplementary material for: Knowledge and social beliefs of malaria and prevention strategies among itinerant Nomadic Arabs, Fulanis and Dagazada groups in Chad: a mixed method study
Source: Malar J. 2022 Feb 19;21:56. doi: 10.1186/s12936-022-04074-0 (PMC8858476; doi:10.1186/s12936-022-04074-0)
Supplement: Supplementary file 3 — Additional file 3: Form: Questionnaire_menage. [file 12936_2022_4074_MOESM3_ESM.docx]

# additional file 3

**Form: Questionnaire_menage**

**1. Questionnaire ménage [household questionnaire]**

**2. Section 1 : consentement, origine ethnique, langue de l'interview**

[Consent, ethnic group, interview language]

**4.Saisir la langue d’interview [write the language used for the survey]**

**5.Sélectionner le groupe d’appartenance de l'interviewée [select the ethnic group]**

Choose one response

- Arabe [Arabic], - Peul [Fulani], - Daza [Daza]

**6. Section 2 : caractéristiques socio-démographiques et économiques [Socio-demographic and economic characteristic]**

**7. Information sur le répondant : [Information on the respondant]**

**8.Quelle est votre relation avec le chef de ménage ? [What is your relationship with the head of household]**

Choose one response

- chef de ménage [head of household], - femme du chef de ménage [wife of the head of houssehold],

- membre du ménage [other member of household], - visiteur [visitor]

**9.Quel est le sexe du répondant ? [What is the gender of respondant]**

Choose one response

- homme [man], - femme [woman]

**10.Quel est son âge ? [How old is the respondant]**

**11.Quelle est votre statut de résidence ? [What is his residence status ?]**

Choose one response

- visiteur [visitor], - resident permanent [permanent resident],

**12.Quel est votre statut matrimonial ? [What is your marital status ?]**

Choose one response

- célibataire [single], - divorcé [divorced], - veuf [widowed], - marié monogame [married monogamy],

- marié polygame [married polygamy]

**13. Information sur le ménage : [Information on the household :]**

**14.Combien d'adultes (plus de 17 ans) vivent dans votre ménage ? [How many adult (older than 17 years old) live in your household?]**

**15.Combien de personnes de 6 - 17 ans vivent dans votre ménage ? [How many persons older 6-17 years live in your household?]**

**16.Combien d'enfant de moins de 6 ans vivent dans votre ménage ? [How many persons older less 6 years live in your household?]**

**17.Quel est le statut matrimonial du chef de ménage ? [What is the marital status of the head of household?]**

Choose one response

- célibataire [single], - divorcé [divorced], - veuf [widowed], - marié monogame [married monogamy],

- marié polygame [married polygamy]

**27. Section 3: connaissances du paludisme et comportement de recherche de soins de santé [Knowledge of malaria and behavior for seeking health]**

**28. Identification du problème de santé : [Health problem identification]**

**29.Quels sont les caractéristiques d’une personne atteinte de paludisme ? [What are characteristics of a person get malaria]**

Choose all that apply

- fièvre [fever], - frisson [shiver], - douleur musculaire [muscle pain], - maux de ventre [stomach], - diarrhee [diarrhea], - nausée [nausea], - vomissement [vomiting], - autre [other]

**30.Quelle est la période de forte incidence du paludisme ? [What is the period of high malaria incidence?]**

Choose all that apply

- début saison sèche (novembre - décembre) [begining of dry season (November-December)], - plein saison sèche (janvier - avril) [dry season (January-April)], - fin saison sèche (mai) [end of dry season (May)], - début saison pluie (juin) [begining of rainy season (June)], - plein saison pluie (juillet - septembre) [rainy season (July-September), - fin saison pluie (octobre) [end of rainy season (October)]

**31.Quels sont les facteurs de risque du paludisme ? [What are risk factors of malaria?]**

Choose all that apply

- eau [water], - chaleur [heat], - moustique [mosquito], - faim [hunger], - nourriture [food], - sort [fate/destiny],

- mystique [mystic], - autre à préciser [other, mentionned]

**32.Quels sont les groupes les plus exposés au paludisme ? [What are groups more at risk of malaria?]**

Choose all that apply

- enfant [children], - femme enceinte [pregnant women], - adultes [adult], - handicapé [disable], - autre à préciser [other, mentionned]

**33.Autres groupes les plus exposés ? [Other groups more at risk of malaria]**

**34. Recours de soins de santé : [Seeking for health :]**

**35.Quel est le premier réflexe pour se faire soigner en cas de paludisme ? [What is your first intention for malaria treatment ?]**

Choose one response

- formation sanitaire [health facility], - médicaments traditionnels [traditional drugs], - vendeurs de médicaments [informal drug seller], - autre à préciser [other, mentionned]

**36.Quel prestataire sollicitez-vous pour les soins ? [Which provider do you seek for health ?]**

Choose one response

- formation sanitaire publique [public health facility], - tradipraticien [traditional healer], - marabout [marabout], - vendeur local [local seller], - formation sanitaire confessionnelle [confessional health facility], - formation sanitaire privée [private health facility], - autre à préciser [other, mentioned]

**37.Autre prestataire ? [Other provider ?]**

**38.Quel type de soignant sollicitez-vous pour les soins ? [Which type of practionner do you seek for health?]**

Choose one response

- médecin [physician], - infirmier [nurse], - marabout [marabout], - vendeur local [local seller], - autre à spécifier [other, mentionned]

**39.Autre soignant ? [other practionner ?]**

**40.Pour quelles raisons ? [For which reasons]**

Choose one response

- degré de la maladie [level of sickness], - coût de soins [cost for treatment], - coût indirect [indirect cost], - soutien [support]

**41.Quelles sont les personnes qui décident du recours au soin ? [Who are decide for health seeking?]**

Choose one response

- chef de menage [head of household], - femme du chef de ménage [wife of head of household], - autre membre du ménage [other household member], - personne externe au ménage [external person to household]

**42.Quelle est votre appréciation de la prestation ? [what is your appreciation of services delivered]**

Choose one response

- très bonne [very good], - bonne [good], - mauvaise [bad], - très mauvaise [very bad]

**43. Section 4: connaissance, couverture et utilisation des méthodes de prévention palustre [knowledge, coverage and use of malaria prevention methods]**

**44. Connaissance de stratégie de prévention : [knowledge of prevention strategies :]**

**45.Quels sont les moyens de protection du paludisme ? [what are means to prevent malaria]**

Choose one response

- MILDA [LLIN], - TPI [ITP], - CPS [SMC], - autre à préciser [Other, mentionned]

**46.Autre protection paludisme ? [Other malaria prevention means ?]**

**47.La MILDA concerne quel groupe d’âge ? [The LLIN target which age group?]**

Choose one response

- enfant de moins de 5 ans [children under 5 years old], - personne de 6 - 17 ans [people 6-17 years old], - adultes [adult], - tout le monde [every person], - femmes enceintes [pregnant women]

**48.Le TPI concerne quel groupe ? [The ITP target which group?]**

Choose one response

- enfant de moins de 5 ans [children under 5 years old], - personne de 6 - 17 ans [people 6-17 years old], - adultes [adult], - tout le monde [every person], - femmes enceintes [pregnant women]

**49.La CPS concerne quel groupe d’âge ? [The SMC target which age group?]**

Choose one response

- enfant de moins de 5 ans [children under 5 years old], - personne de 6 - 17 ans [people 6-17 years old], - adultes [adult], - tout le monde [every person], - femmes enceintes [pregnant women]

**50. Couverture des interventions : [coverage of interventions :]**

**51.Possédez-vous une moustiquaire ? [do you have a mosquito net?]**

Choose one response

- oui [yes], - non [no]

**52.Combien de moustiquaire possédez-vous ? [how many mosquito net do you owned?]**

The answer must be > 0 and < 20

**53.Quel type de moustiquaire possédez-vous ? [which type of mosquito net do you owned]**

Choose all that apply

- MILDA [LLIN], - moustiquaire ordinaire [ordinary mosquito net], - moustiquaire imprégné [impregnated mosquito net]

**54.Quel est l’état des moustiquaires ? [How is the mosquito net status]**

Choose one response

- déchirées et inutilisables [torn and unusable], - trouées et soudées [holed and welded], - trouées [holed], - bonne [good]

**55.Combien de fois avez-vous pris le TPI pendant votre dernière grossesse ? [How many time did you take ITP during your last pregnancy?]**

The answer must be > 0 and < 4

**56.Un de vos enfants ont-ils reçus la CPS ? [Did one of your child received SMC?]**

Choose one response

- oui [yes], - non [no]

**57.Combien de vos enfants ont été traités par la CPS ? [how many of your children received SMC?]**

The answer must be > 0 and < 10

**58.Combien d’enfant possèdent la carte CPS ? [how many of them have the SMC card]**

The answer must be > 0 and < 10

**59.Combien d’enfants ont reçu 4 cycles de CPS ? [how many of them have received 4 SMC cycles?]**

The answer must be > 0 and < 10

**60.Combien d’enfants ont reçu 3 cycles de CPS ? [how many of them have received 3 SMC cycles ?]**

The answer must be > 0 and < 10

**61.Combien d’enfants étaient absents à plus d’un cycle CPS ?[how many children were absent for at least one SMC cycle ?]**

The answer must be > 0 and < 10

**62.Avez-vous refusé la CPS lorsque les distributeurs communautaires sont venus chez vous ? [did you refuse the SMC during the visit of distributors in your household?]**

Choose one response

- oui [yes], - non [no], - pas de visite cps [no SMC visit]

**63. Utilisation pratique des stratégies : [practice use of strategies :]**

**64.Vérifier si la moustiquaire est bien installée ? [check if the mosquito net is installed?]**

Choose one response

- oui [yes], - non [no]

**65.À quelle heure doit-on entrer sous la moustiquaire ? [At what time should sameone enter into net ?]**

**66.Avez-vous passé la nuit dernière sous la moustiquaire ? [did you spent last night under mosquito net?]**

Choose one response

- oui [yes], - non [no]

**67.Combien de dose TPI reçoit-on lors d’une grossesse ? [How many ITP doses can a pregnant women receive during a pregnancy?]**

The answer must be > 0 and < 4

**68.La CPS comprend combien de cycles ? [How many cycles have the SMC?]**

The answer must be > 0 and < 10

**69.Quelle est la posologie de médicaments CPS (vérifier si l’explication est correcte) ? [What is the posology of SMC drugs (check if the answer details is correct)?]**

Choose one response

- oui [yes], - non [no]
